# Effects of carbon nanotubes on intercellular communication and involvement of IL-1 genes

**DOI:** 10.1007/s12079-016-0323-0

**Published:** 2016-04-21

**Authors:** Yke Jildouw Arnoldussen, Kristine Haugen Anmarkrud, Vidar Skaug, Ron N. Apte, Aage Haugen, Shanbeh Zienolddiny

**Affiliations:** Department of Biological and Chemical Work Environment, National Institute of Occupational Health, Pb 8149 Dep, N-0033 Oslo, Norway; The Shraga Segal Department of Microbiology, Immunology and Genetics, The Faculty of Health Sciences, Ben Gurion University of the Negev, 84105 Beer Sheva, Israel

**Keywords:** Intercellular communication, Connexins, CNT, IL-1

## Abstract

An increasing amount of products containing engineered nanoparticles is emerging. Among these particles are carbon nanotubes (CNTs) which are of interest for a wide range of industrial and biomedical applications. There have been raised concerns over the effects of CNTs on human health. Some types of CNTs are classified as group 2B carcinogens by the International Agency for Research on Cancer. CNTs may also induce pulmonary inflammatory and fibrotic effects. By utilizing CNTs of different lengths, we investigated the role of the proinflammatory cytokine, interleukin-1 (IL-1) on gap junctional intercellular communication (GJIC) by using IL-1 wild-type (IL1-WT) and IL-1 knock-out (IL1-KO) cells. GJIC decreased equally in both cell types after CNT exposure. Immunofluorescence staining showed Gja1 and Gjb2 in gap junctions and hemichannels for both cell types. Gjb1 and Gjb2 expression was low in IL1-KO cells, which was confirmed by protein analysis. Gja1 was upregulated with both CNTs, whereas Gjb1 was down-regulated by CNT-2 in IL1-WT cells. Connexin mRNA expression was regulated differently by the CNTs. CNT-1 affected *Gja1* and *Gjb2*, whereas CNT-2 had an effect on *Gjb1*. CNTs negatively affect GJIC through gap junctions independently of the length of CNT and IL-1 status. Furthermore, connexin gene expression was affected by IL-1 at transcriptional and translational levels. As both CNTs used in this study are cytotoxic to the cells and reduce cell survival, we suggest that CNT-induced reduction in GJIC may be important for inhibiting transfer of cell survival signals between cells.

**Electronic supplementary material:**

The online version of this article (doi:10.1007/s12079-016-0323-0) contains supplementary material, which is available to authorized users.

## Background

There is increased focus on the potential negative health effects of exposure to manufactured carbon nanotubes (CNTs). The production and use of CNTs has extensively increased followed by potential health concerns for both work force and consumers (Dong and Ma 2015, Lee et al. 2015). The physico-chemical properties of CNTs such as fibrous structure, high aspect ratio, rigidity and bio-durability are important for the observed inflammagenic, fibrotic and tumorigenic effects (Donaldson et al. 2006, Donaldson et al. 2013, Grosse et al. 2014). In addition, the great structural resemblance to asbestos has significantly increased the focus of understanding the molecular mechanisms underlying the toxicity induced by CNTs (Donaldson et al. 2013).

Inflammation and various signaling pathways may be important for communication between cells (Zhou and Jiang 2014). Intercellular communication is mediated by connexins which are proteins that form hemichannels and gap junction channels between cells. Whereas hemichannels are important for sampling of the extracellular milieu, the gap junction channels are necessary for direct intercellular communication. During the formation of an intercellular channel, six connexin proteins oligomerize into a hemichannel (connexon) and after docking to another hemichannel in an opposing cell the intact channel is formed. Gap junction channels mediate the diffusion of several small and hydrophilic substances including adenosine triphosphate, cyclic adenosine monophosphate, inositol triphosphate, glutathione, glutamate, glucose and several ions between cells (Alexander and Goldberg 2003). Thus far, 21 and 20 different types of connexins have been discovered in humans and mice, respectively, and the permeability and signaling properties of the gap junctions is determined by the specific connexins present (Zhou and Jiang 2014). Gap junctions allow for the transfer of small molecules between adjoining cells and many physiological cellular processes such as proliferation, cell growth and apoptosis are dependent on the transfer through these channels (Alexander and Goldberg 2003, Decrock et al. 2009). Intercellular communication through gap junctions is tightly controlled through rapid dynamic formation and degradation of the gap junctions in response to various types of stimuli including changes in voltage, pH or phosphorylation of connexins (Beardslee et al. 1998, Herve et al. 2007, Lampe and Lau 2004, Falk et al. 2009, Gaietta et al. 2002).

There are only a few studies investigating dysregulation of gap junction intercellular communication (GJIC) as a mechanism of nanoparticle toxicity. In human mesenchymal stem cells the regulation of gap junctions was important for cytotoxicity of quantum dots (Chang et al. 2009). In co-culture of cardiac cells and mesenchymal stem cells, the establishment of GJIC between the two cell types was increased by iron oxide nanoparticles that significantly increased expression of connexin 43 (Cx43) (Han et al. 2015). In human lung A549 cells silver nanoparticles increased GJIC through upregulation of Cx43 protein expression (Deng et al. 2010). Loss of GJIC and delocalization of Cx43 were observed in rat lung epithelial cells exposed to carbon black and silica nanoparticles (Ale-Agha et al. 2010).

CNT exposure of rodents induces inflammation, formation of granulomas and fibrosis (Poland et al. 2008, Porter et al. 2013, Porter et al. 2010, Dong et al. 2015). A recent study showed that elevated expression of the proinflammatory cytokines tumor necrosis factor-α (TNF-α), interleukin-1α (IL-1α), IL-1β, IL-6 and chemokine (C-C motif) ligand 2 (CCL-2) in lung tissues as well as bronchoalveolar lavage from mice exposed to CNTs may play a role (Dong et al. 2015).

Although there is increasing evidence on the role of inflammation in CNT-induced toxicity, fibrogenesis and tumorigenesis (Dong and Ma 2015), the molecular mechanisms remain unknown. A correlation between connexin expression, GJIC and IL-1 proinflammatory cytokines has been reported. For example, treatment of rat astrocytes with a mixture of IL-1β and TNF-α resulted in a reduction in the total and cell surface levels of Cx43 and GJIC (Retamal et al. 2007). These data indicate that regulation of GJIC and connexins depends on the type of nanoparticle, the cell type, and that inflammatory cytokines have a role in this regulation. We have previously investigated the effect of two different CNTs on cellular toxicity including doses from 0.02 μg/ml up to 100 μg/ml (Arnoldussen et al. 2015). The same study showed that CNTs affect several cellular responses and that the IL-1 proinflammatory cytokines may be involved in this (Arnoldussen et al. 2015). Of specific interest was *IL-1α* that was upregulated after CNT exposure (Arnoldussen et al. 2015). Furthermore, a downregulation of precursor IL-1α after 24 h of CNT exposure coincided with a reduction in activated c-Jun N-terminal kinase (JNK) of which IL-1α is an activator. Other studies have shown the importance of GJIC and Cx43 in controlling cell survival and apoptosis (Gilleron et al. 2009, Tekpli et al. 2010). As inflammation, cell survival and apoptosis are linked together after CNT exposure, it was of interest to investigate if GJIC could play a role in these processes. We hypothesized that one of the mechanisms for the CNT effects is through interference with gap junctional intercellular communication in addition to dysregulation of connexin expression. The objective of this study was to investigate i) if CNTs affect intercellular communication ii) if the response of cells to CNTs depends on IL-1 signaling by investigating differences in GJIC in the presence or absence of IL-1 genes.

## Materials and methods

### Cells and cell culture conditions

IL-1α/β wild type (IL1-WT) and IL-1α/β double knock-out (IL1-KO) cell lines were previously described (Arnoldussen et al. 2015, Krelin et al. 2007, Voronov et al. 2010). Briefly, these fibrosarcoma cell lines were derived from 3-methylcholanthrene-induced tumors that were recovered from IL1-WT and IL1-KO BALB/c mice three months after injection of the carcinogen. The same conditions as before were used and passage numbers were kept between 20 and 30. Cells were routinely kept in a humidified 5 % CO_2_ and 95 % air incubator at 37 °C in Dulbecco’s Modified Eagle’s Medium (DMEM, Sigma-Aldrich) containing 10 % fetal bovine serum (FBS, Biochrom), 50 U/ml penicillin and 50 μg/ml streptomycin (Thermo Scientific).

### Characterization of the carbon nanotubes

Two multi-walled carbon nanotubes (CNTs) with different lengths and diameters as described earlier (Arnoldussen et al. 2015) were used; CNT-1 (Mitsui-7 lot #061,220–24) consisted of mainly long fibers (mean length = 5000 nm and mean diameter = 62 nm), CNT-2 consisted of mainly short fibers (mean length = 900 nm and mean diameter = 31 nm). Both CNTs were extensively characterized by SEM, TEM and EDX, and the data are published previously (Arnoldussen et al. 2015).

### Preparation of CNTs for cell culture experiments

Weighing and dispersion were performed as previously described (Arnoldussen et al. 2015). Briefly, CNTs were weighed and added to dispersion media (DM; (Porter et al. 2008)) before sonication on ice (Branson probe sonicator, 30 % amplitude pulse cycle, 3 × 5 min) and immediate addition to cell culture media.

### Functional assay of GJIC by scrape loading

GJIC was determined by quantitative scrape loading (Opsahl and Rivedal 2000). IL1-WT and IL1-KO cells were cultured on cover slips in 12-well plates (NUNC) and grown until 80–90 % confluent. The cells were then exposed to 5 μg/ml of CNT-1 and CNT-2 for 24 h. Before scrape loading the confluent cell layer was washed twice with PBS. Then 1 ml of 0.05 % Lucifer Yellow (Sigma-Aldrich) dissolved in PBS w/o Ca^2+^ and Mg^2+^ was added to each well and the cell monolayer was cut with a surgical scalpel four times. After 4 min the Lucifer Yellow solution was removed, the well was washed with PBS four times and then cells were fixed in 3.7 % formalin o/n. The next day the wells were washed with PBS two times before mounting of the cover slips with Mowiol (Calbiochem). Fluorescence was observed using a laser scanning microscope (LSM 710, Zeiss) with a magnification of 20× and photographs were taken with an AxioCam camera (Zeiss). Ten images were taken for each exposure. Analysis was done by the public domain NIH Image program. The same settings were used for each measurement. The levels of GJIC were analyzed by means of the area of dye-coupled cells.

### Intercellular observation of connexin proteins by immunofluorescence (IF)

IL1-WT and IL1-KO cells were cultured on cover slips and were allowed to attach for 24 h before exposure to 5 μg/ml of CNT-1 and CNT-2 for 24 h. Cells were then fixed for 20 min in 4 % paraformaldehyde and permeabilized with 0.1 % Triton X-100 in PBS for 5 min. Cover slips were blocked by incubation with 5 % BSA in 0.1 % PBS-Triton X-100 for 1 h at room temperature and incubated overnight with a primary antibody against Gja1 (rabbit connexin 43 polyclonal antibody, Cell Signaling Technology), Gjb1 (mouse anti-connexin-32 monoclonal antibody, Millipore) or Gjb2 (goat anti-Gjb2 polyclonal antibody, Abcam) in 3 % BSA in PBS at 4 °C in a humidified chamber. Secondary antibodies anti-rabbit Alexa Fluor 488, anti-mouse Alexa Fluor 488 or anti-goat Alexa Fluor 488 (all from Molecular Probes) were left on the cells for 1 h at room temperature in a humidified chamber. To visualize cell nuclei cells were counterstained with Hoechst (Sigma-Aldrich). Mowiol was used for mounting of the cover slips. Fluorescence was observed using a laser scanning microscope (LSM 710, Zeiss) and pictures were taken with an AxioCam camera (Zeiss).

### Quantitative PCR (qPCR) for measurement of connexin mRNA expression

mRNA levels of *Gja1*, *Gjb1* and *Gjb2* were measured by qPCR. Briefly, total RNA was extracted from IL1-WT and IL1-KO cells using Isol-RNA lysis reagent (5 PRIME) and the obtained RNA was DNase treated with DNA-*free*™ DNA Removal Kit (Ambion). cDNA was then made using qScript cDNA synthesis kit (Quanta Biosciences) according to the manufacturers’ recommendations. qPCR was performed for *Gja1*, *Gjb1*, *Gjb2* and *β-actin* as a housekeeping gene on a StepOne Real-Time PCR system (Applied Biosystems) with Perfecta SYBR Green FastMix, ROX (Quanta BioSciences). Primer sequences are available upon request. A serial diluted internal standard served as a control for the qPCR reaction. Relative gene expression levels were calculated and normalized to *β-actin*.

### Western blot analysis for detection of connexin protein expression

Western blot analysis was performed as described previously (Arnoldussen et al. 2015). Briefly, concentrations of the extracted protein were measured using NanoDrop-8000 (Thermo Scientific). 100 μg of protein for each sample was resolved on AnykD Mini protean TGX stain free gels (Bio-Rad) and transferred to a PVDF membrane (Transblot Turbo Transfer pack, Bio-Rad). The Trans-Blot Turbo blotting system (Bio-Rad) was used for transfer. Antibodies used were as follows; Gja1 (rabbit connexin 43 polyclonal antibody, Cell Signaling Technology), Gjb1 (mouse anti-connexin-32 monoclonal antibody, Millipore), Gjb2 (goat anti-Gjb2 polyclonal antibody, Abcam), Sodium Potassium ATPase alpha 1 (mouse anti-NaK ATPase α1 monoclonal antibody, Abcam) and α-Tubulin (rabbit monoclonal antibody, Cell Signaling Technology). Horseradish peroxidase HRP-conjugated secondary antibodies against rabbit and mouse (both from Cell Signaling Technology) and against goat (Santa Cruz Biotechnology, INC) were used.

### Isolation of integral membrane and membrane-associated proteins

IL1-WT and IL1-KO cells were exposed to 5 μg/ml of CNT-1 and CNT-2 for 24 h. Cells were then harvested and the Mem-PER Plus Membrane Protein Kit (ThermoFisher Scientific) was used to isolate cytosolic and integral membrane/membrane-associated proteins into two fractions. The manufacturers’ instructions were followed precisely. Protein extracts were then subjected to western blot analysis.

### Statistical methods

Statistical analysis of differences in gene and protein expression was performed using the Student’s t-test. *p* < 0.05 were considered as statistically significant.

## Results

**Fig. 1:**
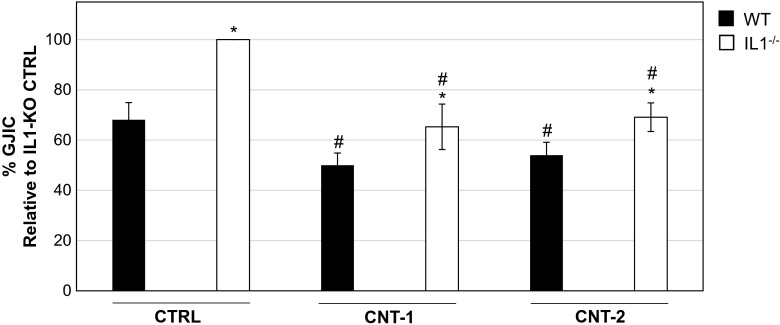
GJIC decreases in IL1-WT and IL1-KO cells after exposure to CNTs. IL1-WT and IL1-KO cells were grown on coverslips and exposed to dispersion media alone as a control or to 5 μg/ml of CNT-1 and CNT-2 for 24 h. After this time scrape loading was performed using Lucifer Yellow. Confocal microscopy was used to detect fluorescence and the levels of GJIC were determined by means of the area of dye-coupled cells. Quantification of three independent experiments is shown where the values represent the mean ± standard error (SE). * *P* < 0.05 between IL1-WT and IL1-KO cells. ^#^ *P* < 0.05 between exposed IL1-WT or IL1-KO and their respective non-exposed controls

Two different CNTs, CNT-1 containing long fibers and CNT-2 containing short fibers, were used. IL1-WT and IL1-KO cells were exposed to 5 μg/ml of each of the two CNTs. Among the different doses tested, this dose reduced the cell number for both CNTs used and had an effect on intracellular signaling, as recently shown (Arnoldussen et al. 2015). The cells’ morphology changed after CNT exposure including increased detachment and a reduction in cellular adhesion (Suppl. Figure 1). Investigation of GJIC showed that it was significantly higher in IL1-KO cells compared to IL1-WT cells both in the control and CNT exposed cells after 24 h (Fig. 1; representative images are shown in Suppl. Figure 2A). Furthermore, exposure by CNT-1 and CNT-2 significantly reduced GJIC compared to controls. The fold reduction compared to controls was similar; approximately 1.5 fold (Fig. 1). Similar results were obtained with CNT exposure for 48 h (Suppl. Figure 2B). Thus, exposure to CNTs leads to a decrease in GJIC, but is not dependent on the presence of IL-1 genes.

**Fig. 2:**
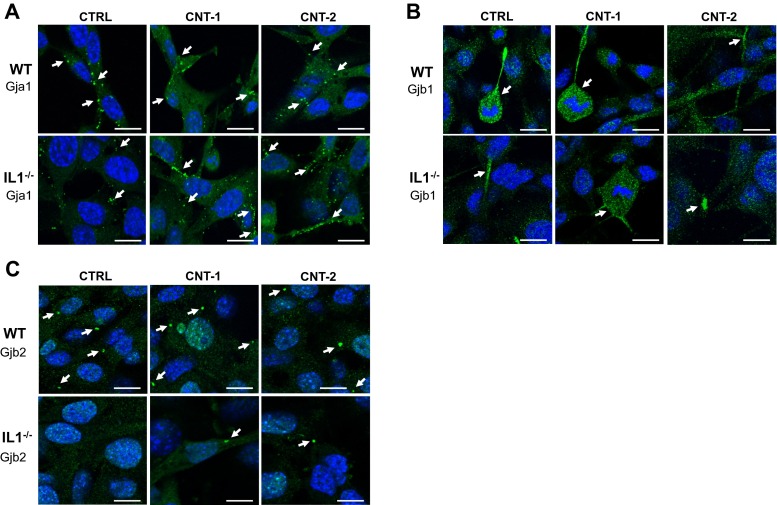
Immunofluorescence images of Gja1, Gjb1 and Gjb2 in IL1-WT and IL1-KO cells after exposure to CNT-1 and CNT-2. IL1-WT and IL1-KO cells were plated on cover slips and were allowed to attach for 24 h before exposure to CNTs or dispersion media alone for 24 h. Cells were stained with the respective antibodies and fluorescent secondary antibodies were used to detect expression. Hoechst staining was used to visualize cell nuclei. **a** Representative images for Gja1. **b** Representative images for Gjb1. **c** Representative images for Gjb2. Arrows indicate interesting areas with hemichannels and gap junction channels when present between cells. For Gjb1 arrows also point to dividing cells having increased expression of Gjb1. Scale bar: 10 μm

Localization of Gja1 (Cx43), Gjb1 (Cx32) and Gjb2 (Cx26) in the cells was studied by immunofluorescence (IF) using laser scanning microscopy. This showed specific staining of Gja1 in hemichannels and gap junctions for both IL1-WT and IL1-KO cells with or without CNT exposure for 24 h (Fig. 2a). Gjb1 was increasingly expressed in mitotic cells and was specifically present in the cells’ protrusions (Fig. 2b). There was, however, a smaller degree of specific localization to hemichannels and gap junctions than observed for Gja1. In addition, staining was low in IL1-KO cells but was independent of CNT exposure (Fig. 2b). Gjb2 was clearly expressed in hemichannels and gap junctions and to a higher extent in IL1-WT cells than IL1-KO cells (Fig. 2c). Again, exposure to CNTs did not affect localization of Gjb2 protein (Fig. 2c).

**Fig. 3:**
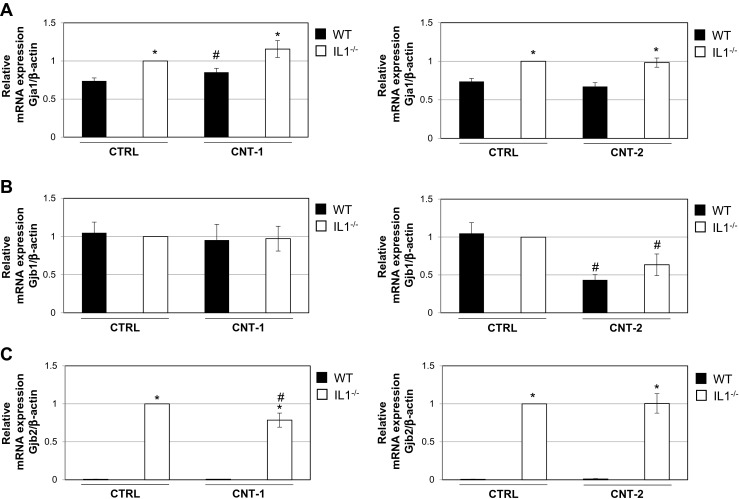
*Gja1*, *Gjb1* and *Gjb2* mRNA expression levels in IL1-WT or IL1-KO cells after exposure to dispersion media alone, CNT-1 or CNT-2**. a** *Gja1* mRNA expression levels investigated by qPCR after exposure to CNT-1 and CNT-2 for 24 h. **b** *Gjb1* mRNA expression levels after exposure to the CNTs for 24 h. **c** *Gjb2* mRNA expression levels after exposure to the CNTs for 24 h. Values represent the mean ± standard error (SE) of three independent experiments performed in triplicate. * *P* < 0.05 between IL1-WT and IL1-KO cells. ^#^ *P* < 0.05 between exposed IL1-WT or IL1-KO and their respective non-exposed controls

To further elucidate GJIC functionality and confirm the IF results, the mRNA expression levels of *Gja1*, *Gjb1* and *Gjb2* were investigated. IL1-WT and IL1-KO cells were exposed to 5 μg/ml of each of the two CNTs for 24 and 48 h. Quantification of their mRNA expression levels as measured by qPCR showed that the two CNTs affected *Gja1*, *Gjb1* and *Gjb2* differently (Fig. 3 and Suppl. Figure 3). *Gja1* expression was significantly increased with CNT-1 in IL1-WT cells compared to control IL1-WT cells. When comparing IL1-WT and IL1-KO cells, *Gja1* expression was significantly increased in IL1-KO cells which was independent of CNT exposure (Fig. 3a). CNT-1 did not have a significant effect on *Gjb1* mRNA expression (Fig. 3b). For CNT-2 exposure, *Gjb1* is significantly reduced for both cell types. Figure 3c shows the results for *Gjb2* mRNA expression. CNT-1 significantly downregulates *Gjb2* in IL1-KO cells compared to the control whereas there is no difference for CNT-2 (Fig. 3c). *Gjb2* expression is significantly increased in IL1-KO cells compared to IL1-WT cells. Thus, the three connexins, *Gja1*, *Gjb1* and *Gjb2*, investigated are regulated differently by the two different CNTs and at least the expression of *Gja1* and *Gjb2* is affected by IL-1.

**Fig. 4:**
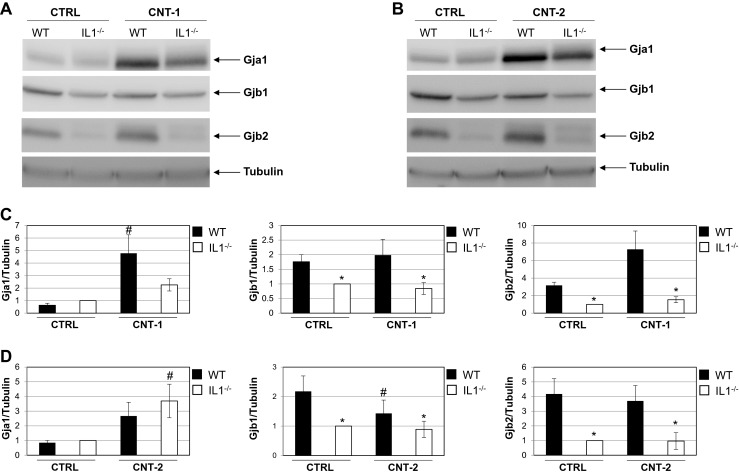
Gja1, Gjb1 and Gjb2 protein levels in IL1-WT or IL1-KO cells after exposure to dispersion media alone, CNT-1 or CNT-2. **a** Representative western blots for Gja1, Gjb1 and Gjb2 after exposure to CNT-1. **b** Representative western blots for Gja1, Gjb1 and Gjb2 after exposure to CNT-2. Tubulin was used as a loading control. **c** Quantification of Gja1, Gjb1 and Gjb2 protein levels normalized to tubulin after exposure to CNT-1. **d** Quantification after exposure to CNT-2. For the bar graphs the values represent the mean ± standard error (SE) of three independent experiments. * *P* < 0.05 between IL1-WT and IL1-KO cells. ^#^ *P* < 0.05 between exposed IL1-WT or IL1-KO and their respective non-exposed controls

The connexin mRNA expression levels were confirmed by western blot analysis of the respective proteins. Representative western blots are shown in Fig. 4a and b after 24 h of CNT exposure. Quantification of the protein levels indicates that CNT-1 increases Gja1 protein expression significantly in IL1-WT cells compared to exposed IL1-WT control cells (Fig. 4c). For CNT-2 a significant increase in Gja1 is observed for IL1-KO cells compared to the control (Fig. 4d). No significant differences for Gja1 between IL1-WT and IL1-KO cells were detected in response to the CNTs (Fig. 4c and d). Gjb1 expression was significantly downregulated in IL1-KO cells compared to IL1-WT cells (Fig. 4c and d). CNT-2 had a greater effect than CNT-1 on Gjb1 as Gjb1 is significantly downregulated in exposed IL1-WT cells compared to IL1-WT control cells (Fig. 4d). Gjb2 expression did not change significantly after CNT exposure (Fig. 4c and d). There was however, a significant downregulation in IL1-KO cells compared to IL1-WT (Fig. 4c and d). Whereas Gja1 expression reduced after 48 h compared to 24 h of exposure (Suppl. Figure 4), Gjb1 and Gjb2 expression did not differ discernibly from the 24 h timepoint (Suppl. Figure 4).

**Fig. 5:**
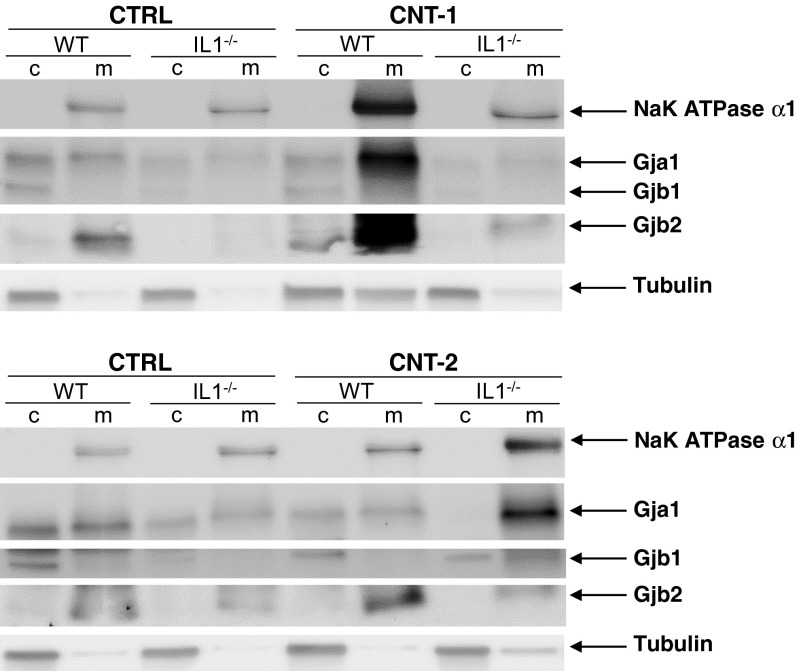
Localization of Gja1, Gjb1 and Gjb2 protein after CNT exposure. Cytosolic (*c*) and integral membrane/membrane-associated (*m*) proteins were separated into two fractions. Western blot analysis was used to investigate the localization of Gja1, Gjb1 and Gjb2. Antibodies against NaK ATPase α1 and α-Tubulin were used to detect the membrane and cytosolic fractions, respectively. The images shown are representative of two independent experiments

As the IF data indicated different localization of the connexins (Fig. 2), fractions containing cytosolic proteins or integrated membrane/membrane-associated proteins were obtained after 24 h of CNT exposure. Western blot analysis showed an increase of Gja1 in the membrane of IL1-WT cells after CNT-1 exposure (Fig. 5). Furthermore, Gja1 expression increased in the membrane of IL1-KO cells with CNT-2. Gjb1 was present in the cytosolic fractions (Fig. 5). Gjb2 was increasingly expressed in the membrane fractions and to a higher extent in IL1-WT cells than IL1-KO cells. Altogether, these findings show that connexins are regulated at the protein level and that IL-1 has a role in this.

## Discussion

Gap junctions and GJIC play an important role in maintaining cellular homeostasis and to communicate processes involved in cell growth, differentiation, cell survival and apoptosis. As nanoparticles affect many of these processes it was of interest to investigate GJIC in response to CNTs and how three different connexins are regulated in IL1-WT and IL1-KO cells after exposure to CNTs.

The CNTs used in this study both have a negative effect on GJIC. We have previously shown that the two CNTs with different physico-chemical properties had different effects on cells (Arnoldussen et al. 2015). We found that CNT-1 had a much greater toxicological potential than CNT-2. This was supported by studies showing that short well-dispersed CNTs such as CNT-2, are less cytotoxic to cells, whereas more agglomerated and tangled longer CNTs such as CNT-1 induced more severe levels of cytotoxicity (Wick et al. 2007, Hamilton et al. 2013). It was therefore surprising to find that the two different CNTs alter GJIC similarly (Fig. 1). The results suggest that CNTs have a negative effect on GJIC by having a direct effect on the outer cell membrane, and that this is independent of their characteristics and induction of intracellular signaling pathways. Notion should be taken that a control experiment including cells treated with a gap junction coupling inhibitor to eliminate the bias of cells absorbing the dye during the process of scraping, was not included. Loss of GJIC has been observed in a few other studies after particle exposure. For example, one study found that exposure of rat lung epithelial cells to carbon black and silica particles induced loss of GJIC and delocalization of Cx43 (Ale-Agha et al. 2010). Furthermore, in rat liver cells, inhibition of GJIC and autophagy were involved in cadmium-induced apoptosis (Zou et al. 2015). These studies have further shown that Cx43 is responsible for the decrease in GJIC. However, they did not compare two types of the same particle having different physico-chemical properties. The levels of apoptosis were low, approximately 1 % in IL1-WT cells after exposure to CNT-1 or CNT-2 (Arnoldussen et al. 2015). However, IL1-KO cells were more robust and had a reduction of 5- and 3-fold in the number of cells undergoing apoptosis after exposure to CNT-1 and CNT-2, respectively, compared to IL1-WT cells after 24 h. This may reflect the higher levels of GJIC in IL1-KO cells, which may give increased transfer of cell survival signals.

To further understand the observed effect of CNTs on GJIC, we investigated the protein expression levels of Gja1, Gjb1 and Gjb2 by IF and western blot analysis. Only Gjb1 was significantly downregulated by CNT-2 and may therefore be part of the decrease observed for GJIC. Gjb2 was present both in hemichannels and gap junctions, but its expression levels were not affected by the CNTs. The permeability of Gjb2 channels is low (Goldberg et al. 2004) and may therefore not have an impact on the decrease in GJIC. Of the three connexins investigated, we observed the highest expression of Gja1 in hemichannels and gap junctions and it is increased, specifically in the cell membrane, after exposure to both CNTs. This does, however, not explain the reduction in GJIC after CNT exposure and other regulatory mechanisms may exist. The short half-life of connexins aids in their highly regulated function and is determined by several events such as phosphorylation, ubiquitylation and sumoylation. For most cell types phosphorylation and ubiquitylation of Cx43 is a signal for its internalization and degradation (Falk et al. 2009, Kjenseth et al. 2010, Leithe et al. 2012, Leithe et al. 2006, Lichtenstein et al. 2011). It is known that connexins form homomeric or heteromeric gap junction channels that typically affect their selective permeability (Goldberg et al. 2004). There is however lack of tools and technologies to distinguish between the different channel types, including determining the functional differences between hemichannels and gap junctions (Bodendiek and Raman 2010, Iyyathurai et al. 2013, Saez and Leybaert 2014, Spray et al. 2006). In addition, there is an emerging understanding of the plethora of non-channel dependent actions of connexins in intracellular signaling (Vinken et al. 2012, Zhou and Jiang 2014). Of the 20 types of connexins currently found in mice, only three were tested here and thus there is the possibility that other connexins play a role in GJIC in the cells which then are degraded in response to CNTs to reduce the levels of GJIC.

Our results indicate that IL-1 is not important for the overall effects on GJIC, but that its presence or absence affects the mRNA and protein expression levels of Gja1, Gjb1 and Gjb2. Both Gjb1 and Gjb2 have decreased staining in IL1-KO cells which was confirmed by western blot analysis and for *Gjb1* mRNA expression after CNT-2 exposure. However, for all exposures IL1-KO cells had a higher basal level of GJIC than IL1-WT. As the IF results show very little Gjb1 in hemichannels and gap junctions (Fig. 2) this protein is probably not important for GJIC in IL1-KO cells. Gjb2 was present both in hemichannels and gap junctions, but as its expression level was significantly lower in IL1-KO cells, it may not have a role in increased GJIC in these cells. Gja1 mRNA and protein expression increases after CNT exposure for 24 h followed with a decrease after 48 h. The increase after 24 h coincides with a decrease in JNK activity especially in the IL1-KO cells after exposure to CNT-1 and CNT-2 (Arnoldussen et al. 2015). Some studies show that the JNK signaling pathway controls Cx43 expression (Kimura and Nishida 2010, Zhang et al. 2013). An increase in Gja1 as observed in our study in both IL1-WT and IL1-KO cells, may account for the low activation of JNK at the 24 h timepoint. TNFα has also been implicated to have a negative effect on Cx43. It was shown to reduce GJIC and Cx43 levels in corneal fibroblasts which was mediated by the JNK signaling pathway (Kimura and Nishida 2010, Kimura et al. 2013). Furthermore, TNFα inhibited Cx43 gap junctions through JNK activity (Zhang et al. 2015, Zhang et al. 2013). TNFα is not expressed in IL1-KO cells (Arnoldussen et al. 2015). In these cells, JNK activity is reduced after 24 h of CNT exposure which coincides with an increase in Gja1 levels (Fig. 4). After 48 h of CNT exposure JNK activity significantly increases (Arnoldussen et al. 2015) concomitant with a reduction in Gja1 (Suppl. Figure 4). Cx43 has been found to directly interact with apoptosis signal-regulating 1 (ASK1) which protected against hydrogen peroxide-induced apoptosis in Cx43-overexpressing rat glioma cells (Giardina et al. 2007). As ASK1 is a direct upstream activator of JNK, an increase in Gja1 as observed in our study after 24 h in both IL1-WT and IL1-KO cells, may account for the low activation of JNK through inhibiting ASK1.

Cx43 has a half-life of between 1.5 and 5 h (Fallon and Goodenough 1981, Laird et al. 1991) and is prone to a high degree of regulation. There is the probability that the half-life of other connexins is regulated in a similar manner. A limitation of this study is the cell type used, the concentrations of the CNTs and the timepoints used. In this regard these factors may be changed and the effects thereof should be investigated in other cell types. Furthermore, it would be of interest to investigate the effects of mature and secreted IL-1 cytokines on GJIC and connexin expression. In the present study, the levels of protein expression were in many instances different from the mRNA expression levels, especially for Gjb2. *Gjb2* was highly expressed in IL1-KO cells and was regulated differently by the CNTs, but low levels of Gjb2 protein were detected in parallel to an increase of Gjb2 in IL1-WT cells. To our knowledge there is no current understanding on the regulation of Gjb2 that could explain these results. However, multiple proteins and other factors may be involved in the regulation of connexins. How changes in these and other pathways influence connexin expression after CNT exposure require further investigation. In conclusion, this is the first study showing that CNTs decrease GJIC independent of their physico-chemical properties. In the cellular response to CNTs the presence or absence of IL-1 does not seem to play an essential role on GJIC.

## Electronic Supplementary Material

Suppl. Figure 1 Cell morphology of IL1-WT or IL1-KO cells after exposure to dispersion media alone, CNT-1 or CNT-2 for 24 h. (PDF 196 kb). Cells were exposed to dispersion media alone or to CNT-1 and CNT-2 and after 24 h images were taken using light microscopy. Representative images are shown

Suppl. Figure 2 GJIC decreases in IL1-WT and IL1-KO cells after exposure to CNTs. IL1-WT and IL1-KO cells were grown on coverslips and exposed to dispersion media alone as a control or to 5 μg/ml of CNT-1 and CNT-2 for 24 and 48 h. After this time scrape loading was performed using Lucifer Yellow. Confocal microscopy was used to detect fluorescence and the levels of GJIC were determined by means of the area of dye-coupled cells. A) Representative images of dye diffusion after scrape loading 24 h after exposure to CNT-1 or CNT-2. Scale bar: 100 μm. B) Quantification of three independent experiments exposed to CNTs for 48 h is shown where the values represent the mean ± standard error (SE). * *P* < 0.05 between IL1-WT and IL1-KO cells. ^#^ *P* < 0.05 between exposed IL1-WT or IL1-KO and their respective non-exposed controls. (PDF 281 kb)

Suppl. Figure 3 mRNA expression levels of *Gja1*, *Gjb1* and *Gjb2* in IL1-WT or IL1-KO cells after exposure to dispersion media alone, CNT-1 or CNT-2 for 48 h. A) *Gja1* mRNA expression levels investigated by qPCR after exposure to CNT-1 and CNT-2 for 48 h. B) *Gjb1* mRNA expression levels after exposure to the CNTs for 48 h. C) *Gjb2* mRNA expression levels after exposure to the CNTs for 48 h. Values represent the mean ± standard error (SE) of three independent experiments performed in triplicate. * *P* < 0.05 between IL1-WT and IL1-KO cells. ^#^ *P* < 0.05 between exposed IL1-WT or IL1-KO and their respective non-exposed controls. (PDF 44 kb)

Suppl. Figure 4 Protein levels of Gja1, Gjb1 and Gjb2 in IL1-WT or IL1-KO cells after exposure to dispersion media alone, CNT-1 or CNT-2 for 48 h. A) Quantification of Gja1, Gjb1 and Gjb2 protein levels normalized to tubulin after exposure to CNT-1 for 48 h. B) Quantification after exposure to CNT-2 for 48 h. Bar graph values represent the mean ± standard error (SE) of three independent experiments. * *P* < 0.05 between IL1-WT and IL1-KO cells. ^#^ *P* < 0.05 between exposed IL1-WT or IL1-KO and their respective non-exposed controls. (PDF 50 kb)
