# Supplementary figures and images for: Effects of carbon nanotubes on intercellular communication and involvement of IL-1 genes
Source: J Cell Commun Signal. 2016 Apr 21;10(2):153–62. doi: 10.1007/s12079-016-0323-0 (PMC4882305; doi:10.1007/s12079-016-0323-0)

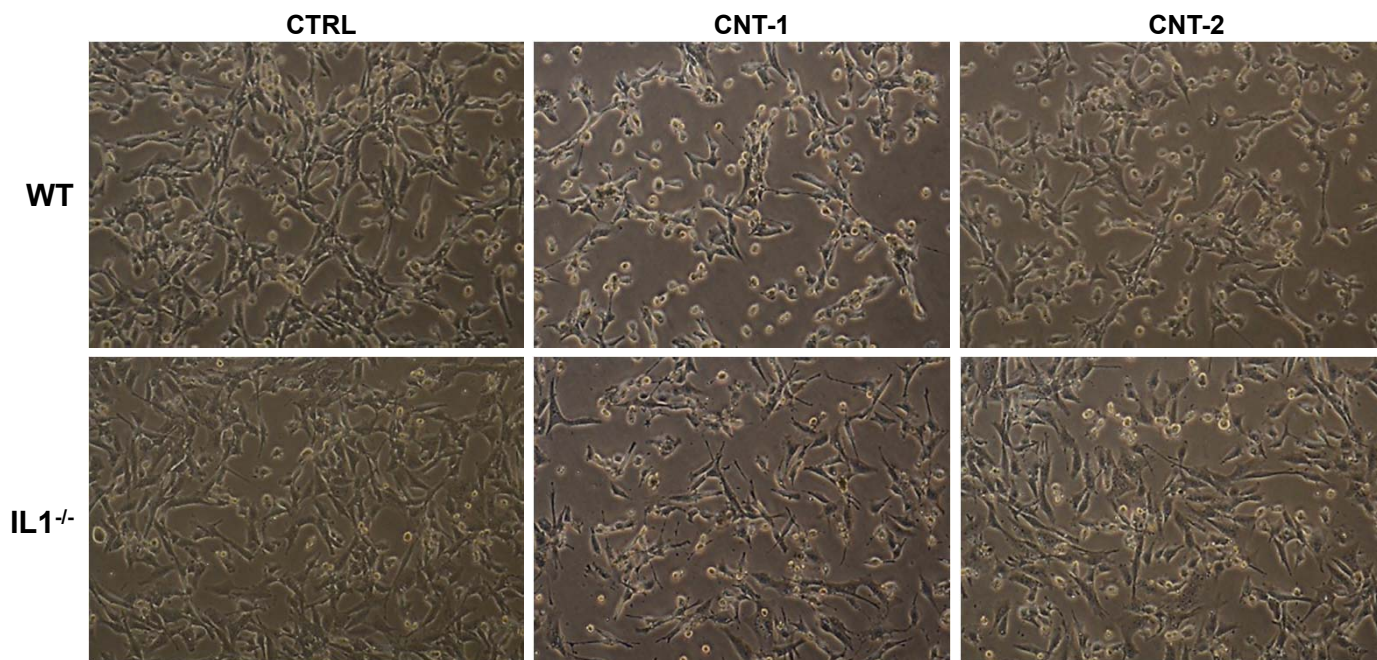

Supplementary Figure 1

Supplement: Supplementary file 1 — Cell morphology of IL1-WT or IL1-KO cells after exposure to dispersion media alone, CNT-1 or CNT-2 for 24 h. (PDF 196 kb). Cells were exposed to dispersion media alone or to CNT-1 and CNT-2 and after 24 h images were taken using light microscopy. Representative images are shown [file 12079_2016_323_MOESM1_ESM.pdf]

**A**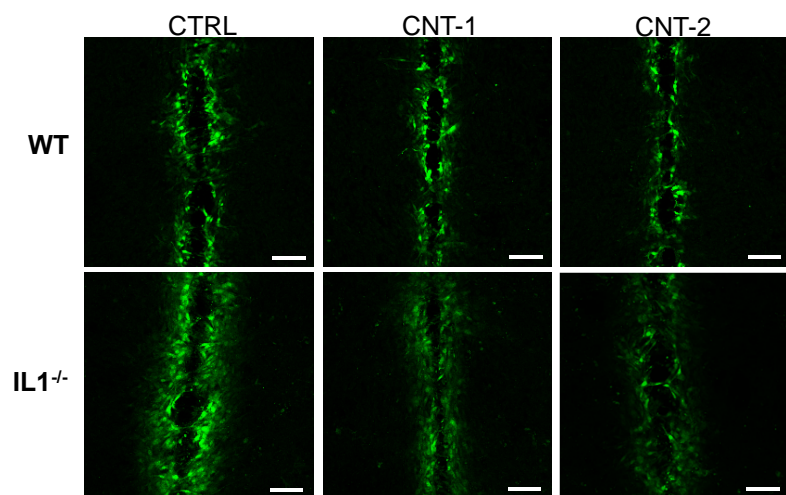**B**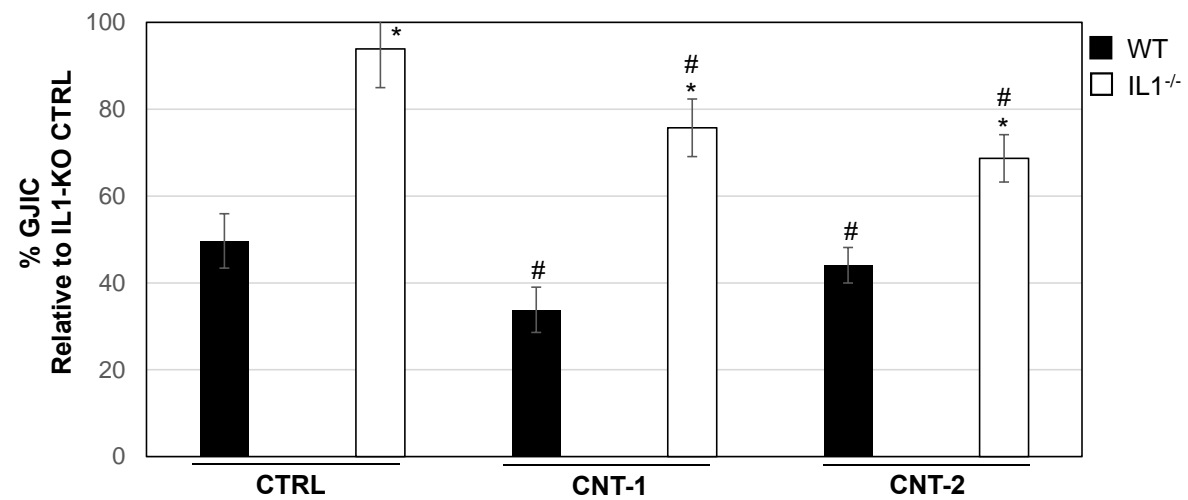

Supplementary Figure 2

Supplement: Supplementary file 2 — GJIC decreases in IL1-WT and IL1-KO cells after exposure to CNTs. IL1-WT and IL1-KO cells were grown on coverslips and exposed to dispersion media alone as a control or to 5 μg/ml of CNT-1 and CNT-2 for 24 and 48 h. After this time scrape loading was performed using Lucifer Yellow. Confocal microscopy was used to detect fluorescence and the levels of GJIC were determined by means of the area of dye-coupled cells. A) Representative images of dye diffusion after scrape loading 24 h after exposure to CNT-1 or CNT-2. Scale bar: 100 μm. B) Quantification of three independent experiments exposed to CNTs for 48 h is shown where the values represent the mean ± standard error (SE). * P < 0.05 between IL1-WT and IL1-KO cells. # P < 0.05 between exposed IL1-WT or IL1-KO and their respective non-exposed controls. (PDF 281 kb) [file 12079_2016_323_MOESM2_ESM.pdf]

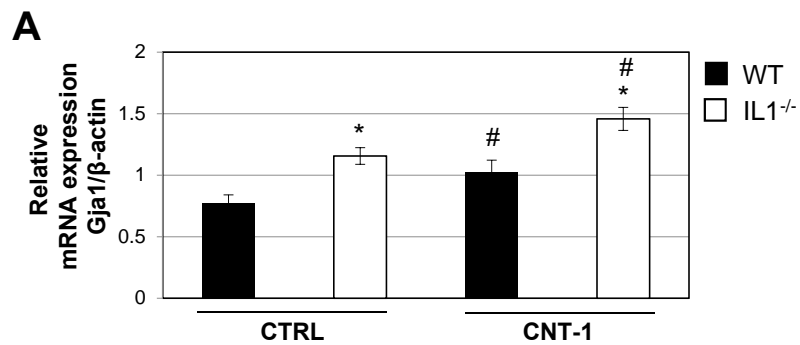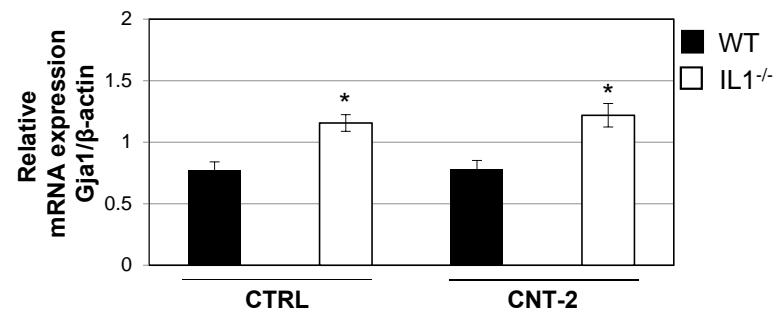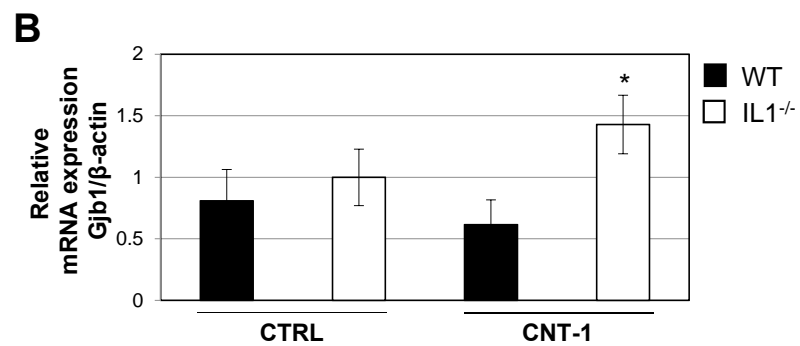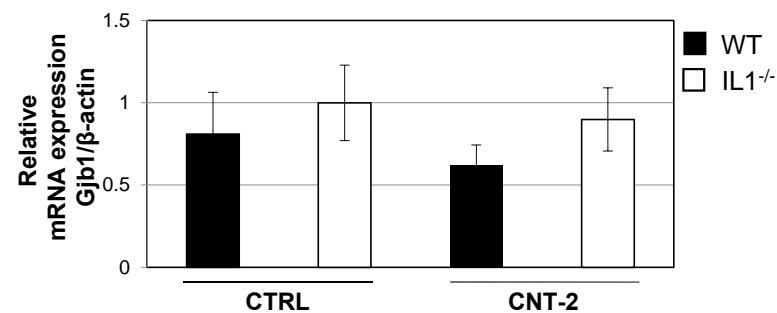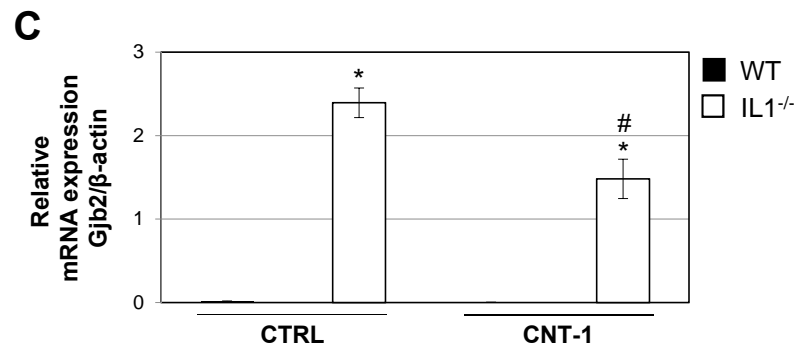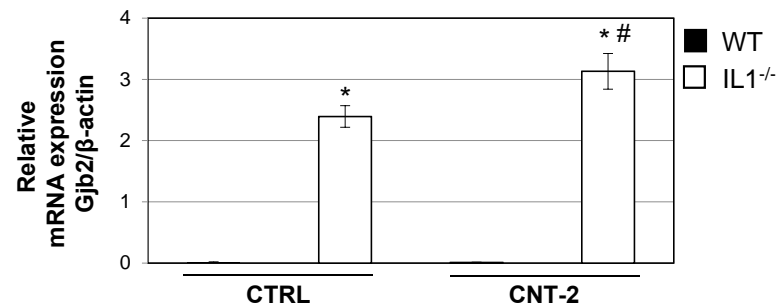

Supplementary Figure 3

Supplement: Supplementary file 3 — mRNA expression levels of Gja1, Gjb1 and Gjb2 in IL1-WT or IL1-KO cells after exposure to dispersion media alone, CNT-1 or CNT-2 for 48 h. A) Gja1 mRNA expression levels investigated by qPCR after exposure to CNT-1 and CNT-2 for 48 h. B) Gjb1 mRNA expression levels after exposure to the CNTs for 48 h. C) Gjb2 mRNA expression levels after exposure to the CNTs for 48 h. Values represent the mean ± standard error (SE) of three independent experiments performed in triplicate. * P < 0.05 between IL1-WT and IL1-KO cells. # P < 0.05 between exposed IL1-WT or IL1-KO and their respective non-exposed controls. (PDF 44 kb) [file 12079_2016_323_MOESM3_ESM.pdf]

**A**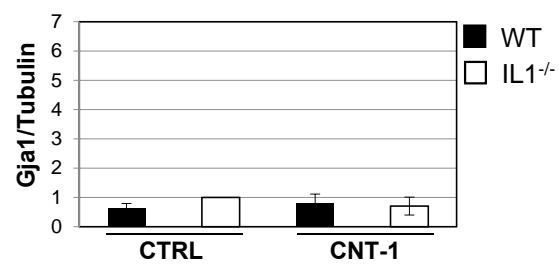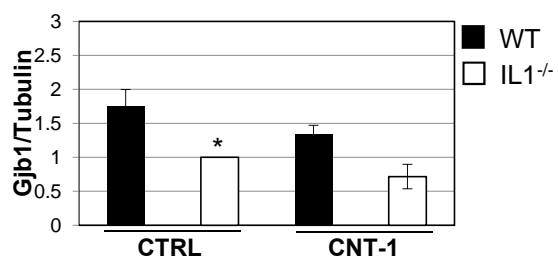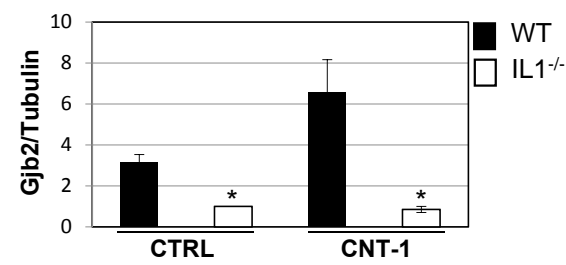**B**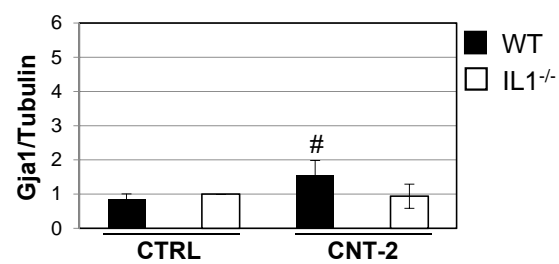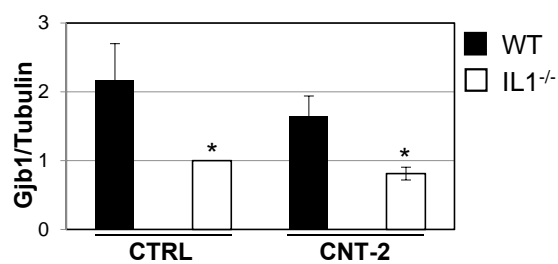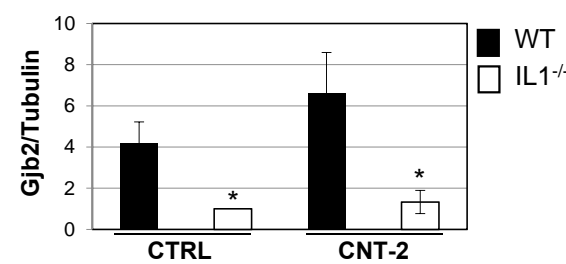

Supplementary Figure 4

Supplement: Supplementary file 4 — Protein levels of Gja1, Gjb1 and Gjb2 in IL1-WT or IL1-KO cells after exposure to dispersion media alone, CNT-1 or CNT-2 for 48 h. A) Quantification of Gja1, Gjb1 and Gjb2 protein levels normalized to tubulin after exposure to CNT-1 for 48 h. B) Quantification after exposure to CNT-2 for 48 h. Bar graph values represent the mean ± standard error (SE) of three independent experiments. * P < 0.05 between IL1-WT and IL1-KO cells. # P < 0.05 between exposed IL1-WT or IL1-KO and their respective non-exposed controls. (PDF 50 kb) [file 12079_2016_323_MOESM4_ESM.pdf]
